# The Imbalanced Patterns and Clinical Significance of Cytokines in Acute Myeloid Leukemia Microenvironment

**DOI:** 10.1002/iid3.70290

**Published:** 2025-11-10

**Authors:** Rong Wang, Keying Jing, Huijuan Zhao, Guoguang Zheng, Jun Cai

**Affiliations:** ^1^ Department of Clinical Laboratory, Henan Provincial People′s Hospital People′s Hospital of Zhengzhou University, Henan University People′s Hospital, School of Clinical Medicine, Henan University Zhengzhou China; ^2^ Henan University People′s Hospital, Department of Clinical Laboratory, Henan Provincial People′s Hospital Henan University Zhengzhou China; ^3^ Basic Medical College Henan University of Science and Technology Luoyang China; ^4^ State Key Laboratory of Experimental Hematology, National Clinical Research Center for Blood Diseases, Haihe Laboratory of Cell Ecosystem, Institute of Hematology & Blood Diseases Hospital Chinese Academy of Medical Sciences & Peking Union Medical College Tianjin China; ^5^ Tianjin Institutes of Health Science Tianjin China

**Keywords:** acute myeloid leukemia, cytokine, imbalanced profile, leukocyte subpopulation, microenvironment

## Abstract

**Introduction:**

Acute myeloid leukemia (AML) is known for its unfavorable prognosis, prompting research efforts of cytokines in the microenvironment to explore new therapeutic targets. Here, we investigated the complex cytokine networks in both AML mice and AML patients to reveal the role of cytokines in AML pathogenesis.

**Methods:**

As a basis for further studies on human, the patterns of cytokines were detected in AML mice by cytokine array panel, and the patterns of cytokines were detected in AML patients by Luminex liquid suspension chip and ELISA. Leukocyte subpopulations in human were analyzed by flow cytometry. Additionally, the associations of cytokine levels with the outcome of AML patients were analyzed by Cox regression analysis, the overall survival curve was assessed by Kaplan‐Meier method. Furthermore, Pearson correlation analysis was used for the correlation analysis of continuous variables.

**Results:**

The imbalanced patterns of cytokines were observed in AML mice. Furthermore, higher levels of CCL3, CCL4 and CXCL10, independent of sex, age, FAB phenotype, patient status and risk molecular, were associated with the poor outcome of AML patients recruited in our study by Cox regression analysis. Additionally, the survival analysis demonstrated that the CCL3^high^ group had a shorter overall survival than the CCL3^low^ group, and a similar result was observed in the analysis of CXCL10. The correlation analysis revealed that Treg cells may be related to the increase of CCL3, while B cell may be associated with for the changes of CXCL10 in AML microenvironment.

**Conclusion:**

The imbalanced patterns of cytokines were observed in both AML mice and AML patients. Interestingly, 3 cytokines (CCL3, CCL4 and CXCL10) were related to the outcome of AML patients, suggesting they are valuable for AML prognosis. Furthermore, the change of leukocyte subpopulations, either as causes or as consequences, may partially account for the change of cytokines in AML condition.

## Graphical abstract

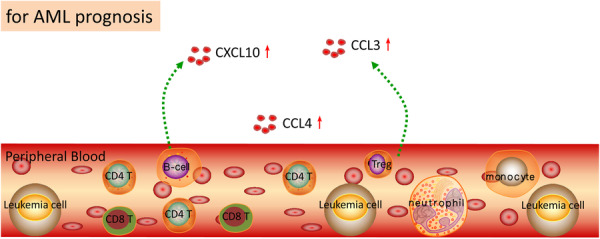

## 1 Introduction

Acute myeloid leukemia (AML) is characterized by high heterogeneity, cytogenetic mutations, aggressive progression, complicated molecular signatures, complex pathogenesis, and a poor clinical outcome [1]. Besides hematopoietic cells themselves, the importance of hematopoietic microenvironment cannot be overemphasized [2]. It is reported that the onset and development of AML is always accompanied by significant remodeling of normal hematopoietic microenvironment into a tumor‐promoting one that supports and protects leukemic stem cells, regardless of the molecular driver mutations triggering the disease [3, 4].

The healthy hematopoietic microenvironment is always kept in a delicate balance and regulated by various components such as immune cell, stromal cells, or signaling molecules including cytokines. It is reported that more M2‐like leukemia‐associated macrophages (LAM) but not total LAMs associated with a poorer prognosis in AML patients, and repolarizing heterogeneous LAMs with more M1 characteristics could eliminate their pro‐leukemic effects [5]. Moreover, the characteristics of bone marrow (BM) and spleen LAM had significant differences in a mouse model with Notch1‐induced T cell acute lymphoblastic leukemia (T‐ALL), suggesting organ‐specific microenvironment could modify the functional and phenotypic characteristics of LAMs [6]. In addition, increased accumulation of more activated regulatory T cells (Treg) was observed in the leukemic hematopoietic microenvironment, and inducible ablation of Tregs prolonged the survival of AML mice by increasing the anti‐leukemic effects of CD8^+^ T cells [7]. Additionally, splenic AML microenvironment strengthened NK cell activation in different stages of leukemia mice, and the function of T cells was regulated by natural killer cells from AML microenvironment. Specifically, the natural killer cells sustained the activation of CD4^+^ T cells, and increased the degranulation of cytotoxic CD8^+^ T cells [8]. Furthermore, AML blasts alter mesenchymal stromal cells activities in the bone marrow niche, favoring disease development and progression, thus aiming at the plasticity of mesenchymal stromal cells to alter the course of acute myeloid leukemia [9].

In patients with pre‐leukemic and leukemic microenvironment, including AML, the precise regulation of cytokines is disrupted, resulting in aberrant cytokine secretion. Studies reported that various cytokines including granulocyte‐macrophage colony‐stimulating factor (GM‐CSF) and different interleukins, are upregulated in AML patient groups [3, 10, 11]. The secretion of hypoxia‐induced IL‐8 by AML cells led to enhanced migration of mesenchymal stem cells into the leukemic BM niche, suggesting the high level of IL‐8 may be associated with poor prognosis in certain AML subsets [3, 12]. In addition, a higher level of MIP‐3β originating from leukemic cells was found to be an accomplice, recruiting T‐ALL cells to the splenic microenvironment in T‐ALL. Furthermore, the AML mice model overexpressing IL‐34, displayed accelerated disease progression, a shorter survival time, and significant subcutaneous invasion of AML cells, suggesting that IL‐34 could accelerate progression of AML [13].

Compared with most cytokines, the levels of TGF‐β and TRAIL are downregulated in the serum of AML patients [3, 14, 15]. TGF‐β1 may be involved in leukemia development by inhibiting AML cell proliferation and survival [15]. Additionally, membrane‐bound macrophage colony‐stimulating factor (M‐CSF) is reported to prolong the survival of AML mice by intrinsically facilitating AML cell differentiation and extrinsically increasing infiltration and phagocytosis of macrophages [16]. Interestingly, IL‐4 is reported to have the potential to suppress the survival of AML cell lines and AML cells derived from patients, regardless of their cytogenetic condition and French‐American‐British (FAB) subtype. The anti‐leukemic effects of IL‐4 at least rely on STAT6 and Caspase‐3 partially, which is consistent with the key role of STAT6 in regulating IL‐4's effects mediating by the IL‐4 receptor [17]. An array of cytokines, together with hematopoietic growth factors, orchestrates the switch from steady‐state hematopoiesis to emergency microenvironment. The AML initiation and progression are triggered by abnormal cytokine and chemokine signaling, which might be promising targets in treatment of AML.

Nevertheless, even though there have been extensive studies on cytokine profiles in AML context, the intricate networks of cytokines in AML pathogenesis remain incompletely understood, due to the high heterogeneity in AML, including various driver mutations, different tumorigenic clones and complex pathogenesis. Therefore, uncovering the molecular basis of the intricate cytokine networks in AML microenvironment is essential to explore new therapeutic alternatives by targeting cytokines as well as their receptors. In this study, we investigated the complex cytokine networks in both AML mice and AML patients, providing clues for clinical diagnosis and treatment.

## 2 Materials and Methods

### 2.1 Mice

Seven to 8 week‐old C57BL/6 J mice were used for the study. All specific pathogen‐free (SPF) mice were purchased and kept in SPF animal facility at the State Key Laboratory of Experimental Hematology. The method for establishing the MLL‐AF9 induced AML mouse model has been described in previous studies [18]. In brief, Lin^‐^ cells, sorted from C57BL/6 J mice, were infected with MSCV‐MLL‐AF9‐GFP retrovirus. Subsequently, the GFP^+^ cells as well as protective cells from healthy bone marrow were transplanted into irradiated C57BL/6 J mice via caudal vein injection. The GFP^+^ leukemia cells, sorted from the primary irradiated AML mice, were transplanted into next generation healthy recipient mice, and the nonirradiated AML mouse model was established. The Animal Care and Use Committee of the Institute of Hematology and Blood Diseases Hospital, CAMS & PUMC, approved all animal procedures involved in this study.

### 2.2 Mouse Cytokine Array

The cytokines and chemokines in supernatant from spleen and bone marrow in healthy or AML mouse were detected by Mouse Cytokine Array Panel A (R&D: ARY006) following the manufacturer′s protocol. The Mouse Cytokine Array Panel A serves as a quick, highly sensitive, and cost‐effective assay to detect various cytokines simultaneously. The supernatant in different groups was the mixture obtained from three mice in respective group to reduce the differences between individuals. The relative changes in cytokine levels between samples were determined by comparing the corresponding signals on different arrays.

### 2.3 Patients and Control Subjects

A total of 91 patients diagnosed with AML were included in this study at Henan Provincial People's Hospital (Zhengzhou, China). In the cytokine assay, 54 cases diagnosed with AML from January 2019 to June 2023 and another 22 healthy individuals (11 males and 11 females; median age, 38; range, 24–65 years) were recruited. The follow‐up of these AML patients was terminated on April 30, 2025. The information of sex, age, FAB phenotype, patient status, risk molecular, was obtained from the electronic medical record system. Additionally, the results of white blood cell (WBC), red blood cell (RBC), hemoglobin (HGB) and blood platelet (PLT) were obtained from laboratory information system when the blood sample were collected. The information of overall survival and outcome was obtained through telephone communication. The subtypes of M2, M3, M4, and M5 were included in FAB phenotype in accordance with the FAB classification system [19]. The patient status included newly‐diagnosed (ND) patients, morphologic partial remission (PR) patients, and AML patients who had undergone allo‐genic hematopoiesis stem cell transplant (allo‐HSCT). The blood samples were collected from ND patients at the first time when they were diagnosed in this hospital. Morphologic PR was evaluated based on International Working Group criteria [20]. The blood samples were obtained from PR patients at the time point when PR patients completed their first evaluation. Additionally, the blood samples were obtained from AML patients who had reestablished hematopoiesis at least 6 months after allo‐HSCT. The preconditioning regimen of allo‐HSCT and the obtainment of stem cells were performed according to the Consensus of Allogeneic Hematopoietic Transplantation for Hematological Diseases in China (2014) [21]. The risk molecular stratification included three levels: good, intermediate, and poor based on different genetic mutations. The good risk group included PML‐RARA, RUNX1‐RUNX1T1, CBFB‐MYH11, *etc*., the intermediate‐risk group included TET2, DNMT3A, and other intermediate risk cytogenetic abnormality, the poor‐risk group included WT1, FLT3‐ITD/TKD, MLL‐AF9, TP53, and other complex cytogenetics.

Additionally, 66 AML subjects were included in the leukocyte subpopulation detection assay. 14 cases were AML‐ND patients (eight males and six females; median age, 53.5; range, 18–73 years), and 52 was in morphologic partial remission (PR) (22 males and 30 females; median age, 49.5; range, 12–72 years). Another 34 healthy individuals (15 males and 19 females; median age, 50.5; range, 12–65 years) were served as the control group.

Furthermore, a total of overlapped 29 AML patients, who were involved in cytokine assay and leukocyte subpopulation assay, were included in the correlation analysis between cytokines and leukocyte subpopulations (14 males and 15 females; median age, 47; range, 16–68 years).

This study was conducted under the guidance of the Ethics Committee of Henan Provincial People's Hospital and written informed consent was obtained from all participants of the study.

### 2.4 Cytokine Detection in Human

We employed Luminex liquid suspension chip (Luminex 200, Millipore, USA) for the detection of 8 cytokines, including CCL3, CCL4, CXCL10, CXCL11, granulocyte colony‐stimulating factor (G‐CSF), IL‐16 IL‐5 and soluble intercellular adhesion molecular‐1 (sICAM‐1), in serum from healthy as well as AML groups. In accordance with the manufacturer's instructions (LXSAHM‐08, R&D, USA), duplicates of the samples were processed. The data were analyzed in accordance with cytokine standard protocols.

Additionally, the expression of CXCL12 in serum was performed by the ELISA kit (R&D:DSA00), following the manufacturer's protocol.

### 2.5 Leukocyte Subpopulation Detection in Human

According to standard protocols, red blood cell lysis buffer was used to treat peripheral blood cells. Subsequently, the cells were marked with antibodies for the determination of their phenotypes (BD). The gating strategies for all subpopulations mentioned in this study were described in detail in the supplementary figures. Notably, neutrophils, monocytes and lymphocytes were gated according to SSC and the expression of CD45. Additionally, CD3^‐^ CD16^+^ CD56^+^ NK cells were gated on the lymphocytes according the expression of CD3 and CD16/CD56, and CD3^‐^ CD19^+^ B cells were gated on the lymphocytes according the expression of CD3 and CD19. Moreover, CD3^+^ T cells were gated on lymphocytes according the expression of CD3, and CD4^+^ T cells and CD8^+^ T cells were gated on CD3^+^ T cells according to the expression of CD4 and CD8 (Figure S2A). Furthermore, CD4^+^ CD25^+^ CD127^low^ Treg cells were gated on CD4^+^ T cells according to the expression of CD25 and CD127 (Figure S2B).

### 2.6 Database Analysis

The TCGA public database was used for the analysis of the overall survival in acute myeloid leukemia patients [22]. Additionally, the method or software version used for quantifying gene expression level was described in the study [22]. We used the statistical software packages R toolkit to find the optimal threshold. Based on the the optimal threshold of mRNA level, the levels of cytokines were divided into the high expression group and the low expression group. Then, we employed the Kaplan‐Meier (K‐M) method to assess the overall survival curve.

### 2.7 Statistical Analysis

We utilized GraphPad Prism 8.0 (San Diego, CA, USA), the statistical software packages R version 3.4.3 (The R Foundation, Vienna, Austria) and SPSS 25.0 (Chicago, IL, USA) for data analysis. Based on the normality of the distribution for continuous variables, continuous variables are presented as mean ± standard deviation (SD) or medians (interquartile range 25%–75% [IQR]), while categorical variables were indicated as frequencies (%). Unpaired Student's t‐test or Mann‐Whitney U‐test depending on the normality of the distribution was used to analyze the comparisons between two groups, whereas one‐way ANOVA was performed to analyze the comparisons of multiple groups, and chi‐square test for categorical variables. Hazard ratios (HRs) and 95% CIs were calculated for AML outcome with variables using Cox proportional hazards models. In the multivariate‐adjusted models, the crude model is the non‐adjusted model with no covariates adjusted. AdjustedⅠmodel is the minimally‐adjusted model with sex and age. Adjusted Ⅱ model is adjusted with sex, age and FAB phenotype. Adjusted Ⅲ model is the fully‐adjusted model with covariates adjusted (sex, age, FAB phenotype, patient status, risk molecular). The subgroup analyses were performed using stratified Cox regression models. Age, a continuous variable, was converted to a categorical variable according to the elderly (60 years) and the mean of age in this cohort (44 years), and then performed an interaction test. Interaction across subgroups was tested using the likelihood ratio test. We used the statistical software packages R toolkit to find the optimal threshold of the cytokine level. Based on the the optimal threshold of cytokine level, the cytokines were divided into the high expression group and the low expression group. Then, the overall survival curve was assessed by Kaplan‐Meier method. Furthermore, Pearson correlation analysis was used for the correlation analysis of continuous variables. *p* < 0.05 was considered statistically significant.

## 3 Results

### 3.1 The Patterns of Cytokines in AML Mice

**Figure 1:**
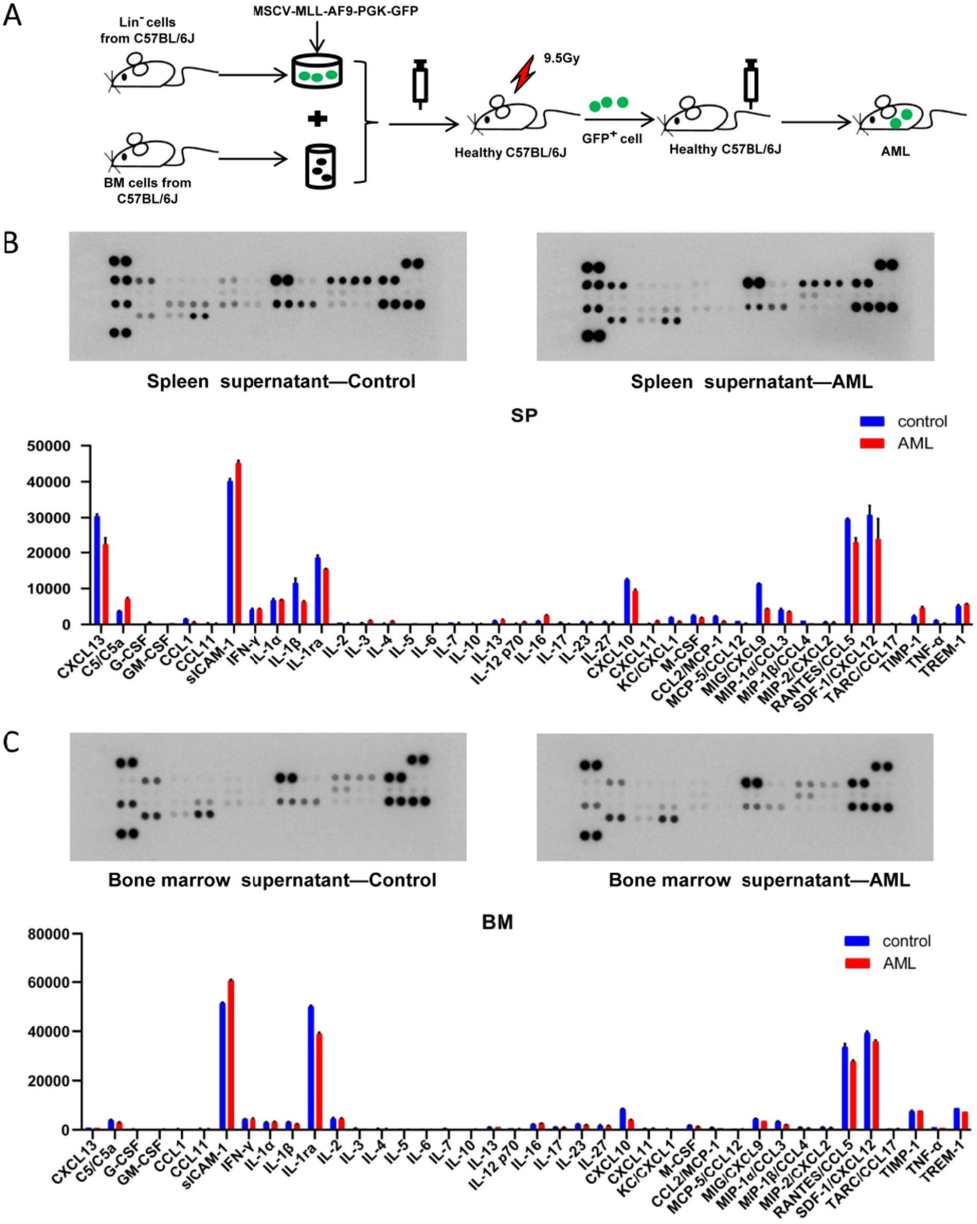
The patterns of cytokines in AML mice. (A) The nonirradiated AML mouse model was established as described. (B) The cytokines and chemokines in supernate from spleen were described (C) The cytokines and chemokines in supernate from bone marrow were described.

The nonirradiated AML mouse model was established as described in the previous article [5, 6, 7] and Materials Methods (Figure 1A). The cytokines and chemokines in supernatant from spleen and bone marrow in healthy or AML mouse were detected, the level of these cytokines is showed in Figures 1B and 1C. The changes in the cytokine panel are summarized in Table S1. Notably, compared with the healthy control group, the level of 5 cytokines (CXCL11, G‐CSF, IL‐16, IL‐5 and sICAM‐1) in both spleen and bone marrow was higher in AML group. However, the level of 22 cytokines (IL‐17, CCL1, CCL11, CCL2/MCP‐1, CXCL10, CXCL13, IL‐10, IL‐1β, IL‐1rα, IL‐7, IL‐23, IL‐27, CCL12/MCP‐5, M‐CSF, CXCL9/MIG, CCL3/MIP‐1α, CCL4/MIP‐1β, CXCL2/MIP‐2, CCL5/RANTES, CXCL12/SDF‐1, CCL17/TARC and TNF‐α) in both spleen and bone marrow was lower in AML group, suggesting the imbalanced profile of cytokines in AML microenvironment in mice.

### 3.2 The Patterns of Cytokines in AML Patients

Based on the above results in AML mice and previous literatures, a total of nine candidate cytokines, including 5 cytokines (CXCL11, G‐CSF, IL‐16, IL‐5 and sICAM‐1), whose expressions were higher in both spleen and bone marrow of AML group, and another 4 cytokines reported in previous research that might be involved in AML progression (CCL3, CCL4, CXCL10 and CXCL12) [7, 23, 24], were selected for further investigation in AML patients. Their expressions were lower in both spleen and bone marrow of AML group in our study. Moreover, the expression levels are relatively high and easily detected.

**Figure 2:**
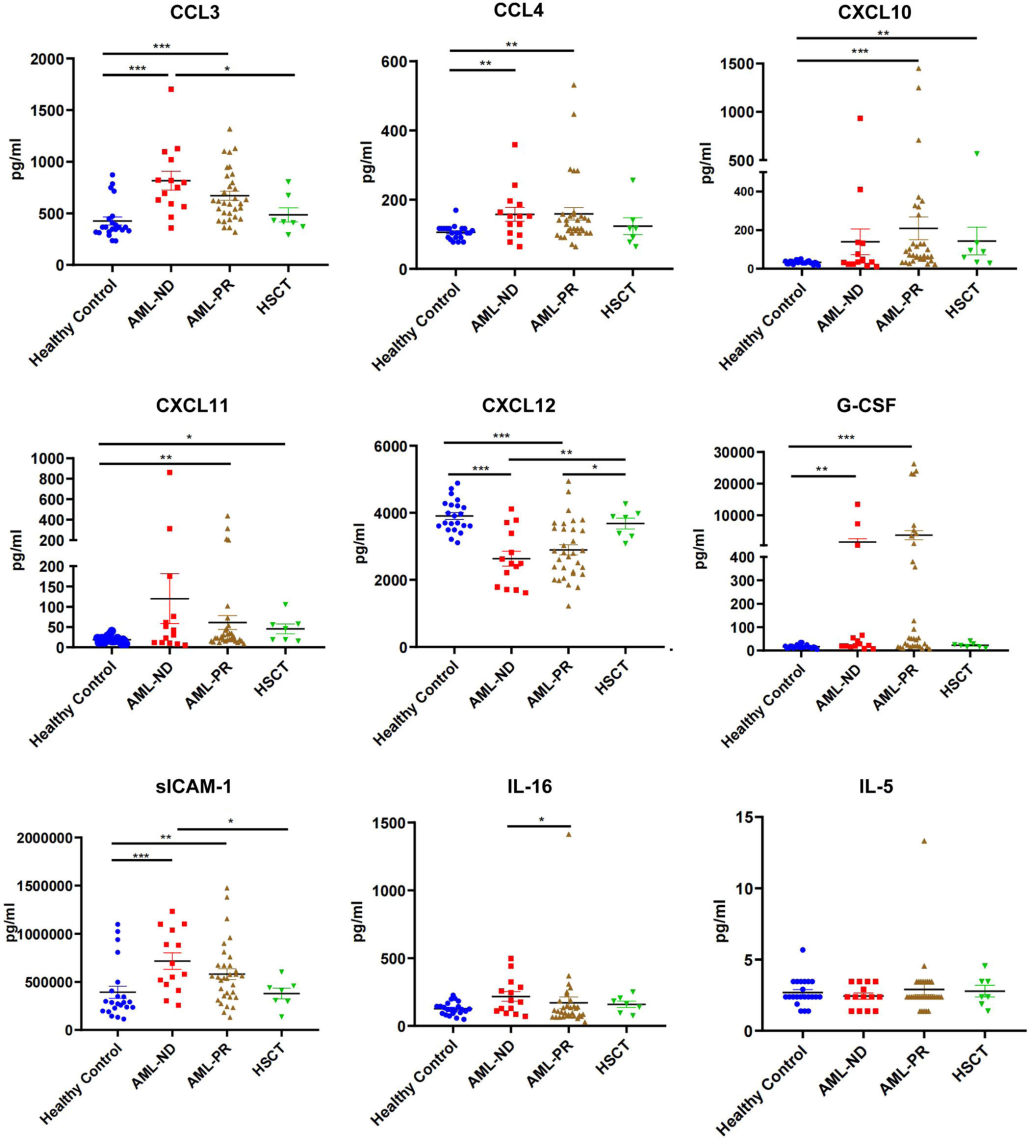
The patterns of cytokines in AML patients. A total of nine candidate cytokines were selected for further verification in AML patients, and the AML patients in this assay were divided into three subgroups, the newly diagnosed group (AML‐ND), the partial remission group (AML‐PR) and the hematopoietic stem cell transplantation patients (HSCT). Data in columns are shown as the mean ± SEM. (*p < 0.05, **p < 0.01, and ***p < 0.001.).

The AML patients in this assay were divided into three subgroups, the newly diagnosed group (AML‐ND), the partial remission group (AML‐PR) and the AML patients who had reestablished hematopoiesis after allo‐HSCT (HSCT). The results of cytokine detection showed that compared to the healthy control group, the serum levels of CCL3, CCL4, G‐CSF and sICAM‐1 were significantly higher in both AML‐ND and AML‐PR groups (Figure 2), and an opposite result was observed in the expression of CXCL12, suggesting that CCL3, CCL4, G‐CSF, sICAM‐1 and CXCL12 may be helpful for AML diagnosis. Furthermore, the levels of CXCL10 and CXCL11 in both AML‐PR and HSCT groups were significantly higher than those in the healthy control group, indicating that the increase of CXCL10 and CXCL11 in AML‐PR and HSCT patients may be associated with AML therapy. Additionally, the level of IL‐16 in AML‐PR group was lower than that in AML‐ND group, suggesting that IL‐16 may be helpful for AML remission. The significant lower level of CCL3, sICAM‐1, and the higher level of CXCL12, in HSCT group than those in AML‐ND group, indicated that CCL3, sICAM‐1 and CXCL12 may be related to the reestablished hematopoiesis after HSCT. However, there was no significant difference of the IL‐5 level among these groups.

Similar results were observed in the expression of G‐CSF, sICAM‐1 and CXCL12 in both AML mice and AML patients. However, the remaining results in AML patients that are not completely consistent or even opposite with that in AML mice, suggest the heterogeneity among species about the imbalanced profile of cytokines in AML microenvironment.

### 3.3 The Association Between the Outcome and the Cytokine Expression in AML Patients

A total of 54 cases diagnosed with AML were recruited in the cytokine assay, we conducted a retrospective study based on this cohort. The follow‐up of these AML patients was terminated on April 30, 2025. The demographic and clinical characteristics of these AML patients are demonstrated in the Table 1. Among the 54 participants from the study, the outcome of 9 subjects was death and 45 subjects survived. The ratio of male to female is equal (27 VS 27), each accounting for 50%, and the median age of all cases was 45.5 years. However, there was no significant difference between the survival group and death group about these variables.

**Table 1:** Demographic and clinical characteristics of the AML patients with cytokine detection.

Variables	Total (*n* = 54)	Survival (*n* = 45)	Death (*n* = 9)	*p*
Sex, *n* (%)				0.467
Female	27 (50.0)	21 (46.7)	6 (66.7)	
Male	27 (50.0)	24 (53.3)	3 (33.3)	
Age (year), Median (IQR)	45.5 (33.2, 52.8)	41.0 (32.0, 51.0)	51.0 (40.0, 53.0)	0.227
FAB Phenotype, *n* (%)				0.891
M2	33 (61.1)	27 (60)	6 (66.7)	
M3	1 (1.9)	1 (2.2)	0 (0)	
M4	16 (29.6)	14 (31.1)	2 (22.2)	
M5	4 (7.4)	3 (6.7)	1 (11.1)	
Patient status, *n* (%)				0.601
ND	14 (25.9)	11 (24.4)	3 (33.3)	
PR	33 (61.1)	27 (60)	6 (66.7)	
HSCT	7 (13.0)	7 (15.6)	0 (0)	
Risk Molecular, n (%)				0.427
Good	16 (29.6)	14 (31.1)	2 (22.2)	
Intermediate	19 (35.2)	17 (37.8)	2 (22.2)	
Poor	19 (35.2)	14 (31.1)	5 (55.6)	
OS (month), Mean ± SD	28.8 ± 20.6	31.0 ± 21.6	18.2 ± 8.7	0.09
CCL3 (pg/mL), Median (IQR)	620.7 (463.4, 814.2)	598.3 (462.6, 798.0)	944.8 (594.7, 1126.0)	0.095
CCL4 (pg/mL), Median (IQR)	128.8 (104.0, 158.2)	128.8 (104.0, 152.4)	152.4 (116.6, 283.5)	0.218
CXCL10 (pg/mL), Median (IQR)	66.9 (36.4, 131.9)	65.8 (33.5, 129.1)	129.7 (60.4, 409.4)	0.084
CXCL11 (pg/mL), Median (IQR)	26.5 (16.2, 55.1)	22.7 (15.2, 45.9)	51.7 (22.7, 72.7)	0.201
CXCL12 (pg/mL), Median (IQR)	2837.0 (2273.0, 3639.0)	2860.0 (2360.0, 3535.0)	2814.0 (2209.0, 3674.0)	0.944
G‐CSF (pg/mL), Median (IQR)	28.4 (16.2, 299.9)	28.4 (15.9, 357.3)	30.0 (22.1, 127.4)	0.5
sICAM‐1 (pg/mL), Median (IQR)	537100.0 (362200.0, 687600.0)	535100.0 (385600.0, 692300.0)	548600.0 (338100.0, 670900.0)	0.954
IL‐16 (pg/mL), Median (IQR)	132.5 (83.2, 228.6)	135.7 (92.0, 205.5)	85.6 (65.1, 257.2)	0.437
IL‐5 (pg/mL), Median (IQR)	2.4 (2.4, 3.5)	2.4 (2.4, 3.5)	2.4 (2.4, 3.5)	0.767
WBC (10^9^/L), Median (IQR)	3.4 (1.4, 5.0)	3.4 (1.4, 5.0)	3.8 (2.2, 4.9)	0.651
RBC (10^12^/L), Median (IQR)	2.5 (2.2, 3.6)	2.4 (2.1, 3.6)	3.0 (2.2, 3.3)	0.917
HGB (g/L), Median (IQR)	79.0 (67.5, 111.0)	77.0 (67.0, 112.0)	95.0 (70.0, 105.0)	0.972
PLT (10^9^/L), Median (IQR)	57.0 (24.2, 127.8)	59.0 (24.0, 121.0)	52.0 (27.0, 164.0)	0.816

*Note: p* < 0.05 indicated a statistically significant difference.

Abbreviations: HGB, hemoglobin; HSCT, allo‐genic hematopoiesis stem cell transplant; IQR, interquartile range; ND, newly‐diagnosed; OS, overall survival; PLT, platelet; PR, partial remission; RBC, red blood cell; WBC, white blood cell.

The univariate cox regression models in Table 2 showed the HRs and 95% CIs for risk of death outcome in AML patients by different variables. The results indicated that the levels of CCL3 (95% CIs 1, 1.0033), CCL4 (95% CIs 1, 1.0064) and CXCXL10 (95% CIs 1, 1.0017), but not other variables, were associated with the risk of death outcome in AML cases.

**Table 2:** Univariate cox regression models for the AML patients with cytokine expression.

Variables	HR (95%CI)	*p* value
Sex:		
Female	Ref	
Male	0.47 (0.12,1.89)	0.289
Age	1.03 (0.97,1.08)	0.321
FAB Phenotype:		
M2	Ref	
M3	0 (0, Inf)	0.999
M4	0.68 (0.14,3.37)	0.635
M5	1.06 (0.13,8.79)	0.959
Patient status:		
ND	Ref	
PR	0.49 (0.12,1.98)	0.318
HSCT	0 (0, Inf)	0.999
Risk molecular		
Good	Ref	
Intermediate	0.98 (0.14,7.02)	0.987
Poor	2.18 (0.42,11.27)	0.354
CCL3 (pg/mL)	1.0033 (1.001,1.0056)	0.004
CCL4 (pg/mL)	1.0064 (1.0003,1.0125)	0.04
CXCL10 (pg/mL)	1.0017 (1.0002,1.0031)	0.022
CXCL11 (pg/mL)	1.0022 (0.9977,1.0067)	0.335
CXCL12 (pg/mL)	1 (0.9992,1.0009)	0.935
G‐CSF (pg/mL)	0.9999 (0.9997,1.0001)	0.517
sICAM‐1 (pg/mL)	1 (1,1)	0.843
IL‐16 (pg/mL)	1.0019 (0.9999,1.0039)	0.064
IL‐5 (pg/mL)	1.14 (0.92,1.42)	0.223
WBC (10^9^/L)	1.02 (0.76,1.37)	0.88
RBC (10^12^/L)	0.88 (0.41,1.87)	0.73
HGB (g/L)	0.993 (0.9685,1.018)	0.578
PLT (10^9^/L)	1.0002 (0.9943,1.0062)	0.942

*Note:*Data presented are HRs and 95% CIs. *p* < 0.05 was considered statistically significant.

Abbreviation: Ref, reference.

The multivariable cox regression models in Table 3 demonstrated the HRs and 95% CIs for risk of AML death by the expression of CCL3, CCL4, and CXCL10, respectively. The crude model is the non‐adjusted model with no covariates adjusted. Adjusted Ⅰ model is the minimally‐adjusted model with sex and age. Adjusted Ⅱ model is adjusted with sex, age and FAB phenotype. Adjusted Ⅲ model is the fully‐adjusted model with covariates adjusted (sex, age, FAB phenotype, patient status, risk molecular). The results indicated that the associations of CCL3 expression, CCL4 expression or CXCL10 expression with the outcome of AML patients were stable in all adjusted models (*p* < 0.05). The increase in the expression of these three cytokines (CCL3, CCL4 or CXCL10) will increase the risk of AML death.

**Table 3:** Multivariable cox regression models for the AML patients with cytokine expression.

Variables	N.(%)	Crude HR (95% CI)	Adjusted Ⅰ HR (95% CI)	Adjusted Ⅱ HR (95% CI)	Adjusted Ⅲ HR (95% CI)
CCL3	9/54(16.7)	1 (1 ~ 1.01)	1 (1 ~ 1.01)	1 (1 ~ 1.01)	1 (1 ~ 1.01)
*p* value		0.004	0.009	0.003	0.038
CCL4		1.01 (1 ~ 1.01)	1.01 (1 ~ 1.01)	1.01 (1 ~ 1.01)	1.01 (1 ~ 1.02)
*p* value		0.04	0.027	0.029	0.024
CXCL10		1 (1 ~ 1)	1 (1 ~ 1)	1 (1 ~ 1)	1 (1 ~ 1)
*p* value		0.022	0.036	0.004	0.008

*Note:*Data presented are HRs and 95% CIs. The crude model is the non‐adjusted model with no covariates adjusted. AdjustedⅠmodel is the minimally‐adjusted model with sex and age. Adjusted Ⅱmodel is adjusted with sex, age and FAB phenotype. Adjusted Ⅲ model is the fully‐adjusted model with covariates adjusted (sex, age, FAB phenotype, patient status, risk molecular). *p* < 0.05 was considered statistically significant.

Abbreviation: Ref, reference.

We did stratified analyses and interactive analyses to see whether the association between cytokine expression and the outcome of AML was stable or not in different subgroups (Tables 4, 5, 6). The results of Table 4 showed that the associations between CCL3 expression and the death outcome of AML were stronger for the participants who were younger (< 60 years, *p* = 0.001, or < 44 years, *p* = 0.024). Furthermore, the associations were stronger for female participants (*p* = 0.006). The results of Table 5 showed that age played an interactive role in the association between CCL4 expression and the death outcome of AML (44 years, *p* for interaction = 0.004). In addition, the associations were stronger for the participants who were younger (< 60 years, *p* = 0.023, or < 44 years, *p* = 0.004). Furthermore, the associations were stronger for male participants (*p* = 0.034). The results of Table 6 showed that the associations were stronger for the participants who were 44–60 years (< 60 years, *p* = 0.016, or > 44 years, *p* = 0.044). Furthermore, the associations were stronger for M2 participants (*p* = 0.014) and PR patients (*p* = 0.008).

**Table 4:** Subgroup analyses for the AML patients according the expression of CCL3.

Subgroup	HR 95CI	*p* value	*p* for interaction
Age			
< 60	1 (1 ~ 1.01)	0.001	0.999
≥ 60	0.87 (0 ~ 5.42160287110597e + 86)	0.999	
< 44	1 (1 ~ 1.01)	0.024	0.125
≥ 44	1 (1 ~ 1)	0.336	
Sex			
Female	1 (1 ~ 1.01)	0.006	0.182
Male	1 (1 ~ 1.01)	0.919	
FAB Phenotype			
M2	1 (1 ~ 1)	0.385	0.999
M3	1 (1 ~ 1)	NA	
M4	1.06 (0 ~ 3.43003424676524e + 74)	0.999	
M5	1.08 (0 ~ 1.18089482649778e + 88)	0.999	
Patient status:			
ND	1.7 (0 ~ 1.66661320546008e + 83)	0.996	0.995
PR	1 (1 ~ 1)	0.675	
HSCT	1 (1 ~ 1)	NA	
Risk molecular			
Good	1 (1 ~ 1.01)	0.509	0.938
Intermediate	1 (1 ~ 1.01)	0.135	
Poor	1 (1 ~ 1.01)	0.055	

*Note:*Data presented are HRs and 95% CIs. Age, a continuous variable, was converted to a categorical variable according to the elderly (60 years) and the mean of age in this cohort (44 years), and then performed an interaction test. Interaction across subgroups was tested using the likelihood ratio test. *p* < 0.05 was considered statistically significant.

Abbreviation: Ref, reference; NA, not applicable.

**Table 5:** Subgroup analyses for the AML patients according the expression of CCL4.

Subgroup	HR 95CI	*p* value	*p* for interaction
Age			
< 60	1.01 (1 ~ 1.01)	0.023	0.998
≥ 60	0.59 (0~Inf)	0.999	
< 44	1.02 (1.01 ~ 1.04)	0.004	0.004
≥ 44	1 (0.99 ~ 1.01)	0.924	
Sex			
Female	1 (0.99 ~ 1.01)	0.417	0.22
Male	1.02 (1 ~ 1.03)	0.034	
FAB Phenotype			
M2	1 (1 ~ 1.01)	0.204	0.999
M3	1 (1 ~ 1)	NA	
M4	1.01 (1 ~ 1.03)	0.094	
M5	5.41 (0~Inf)	0.999	
Patient status:			
ND	1.01 (0.99 ~ 1.03)	0.167	1
PR	1.01 (1 ~ 1.01)	0.13	
HSCT	1 (1 ~ 1)	NA	
Risk molecular			
Good	1.01 (1 ~ 1.02)	0.094	0.636
Intermediate	1.01 (0.99 ~ 1.02)	0.294	
Poor	1 (0.99 ~ 1.02)	0.698	

*Note:* Data presented are HRs and 95% CIs. Age, a continuous variable, was converted to a categorical variable according to the elderly (60 years) and the mean of age in this cohort (44 years), and then performed an interaction test. Interaction across subgroups was tested using the likelihood ratio test. *p* < 0.05 was considered statistically significant.

Abbreviations: NA, not applicable; Ref, reference.

**Table 6:** Subgroup analyses for the AML patients according the expression of CXCL10.

Subgroup	HR 95CI	*p* value	*p* for interaction
Age			
− < 60	1 (1 ~ 1)	0.016	0.997
≥ 60	0.14 (0~Inf)	0.997	
< 44	1 (0.99 ~ 1.01)	0.682	0.5
≥ 44	1 (1 ~ 1)	0.044	
Sex			
Female	1 (1 ~ 1)	0.18	0.479
Male	1 (1 ~ 1.01)	0.054	
FAB Phenotype			
M2	1 (1 ~ 1)	0.014	0.467
M3	1 (1 ~ 1)	NA	
M4	1 (1 ~ 1)	0.143	
M5	1 (0.99 ~ 1.01)	0.797	
Patient status:			
ND	1 (1 ~ 1)	0.95	1
PR	1 (1 ~ 1)	0.008	
HSCT	1 (1 ~ 1)	NA	
Risk molecular			
Good	1 (1 ~ 1.01)	0.124	0.373
Intermediate	1.02 (0.98 ~ 1.07)	0.327	
Poor	1 (1 ~ 1)	0.394	

*Note:* Data presented are HRs and 95% CIs. Age, a continuous variable, was converted to a categorical variable according to the elderly (60 years) and the mean of age in this cohort (44 years), and then performed an interaction test. Interaction across subgroups was tested using the likelihood ratio test. *p* < 0.05 was considered statistically significant.

Abbreviations: NA, not applicable; Ref, reference.

### 3.4 The Overall Survival Analysis in AML Patients by the Cytokine Expression

**Figure 3:**
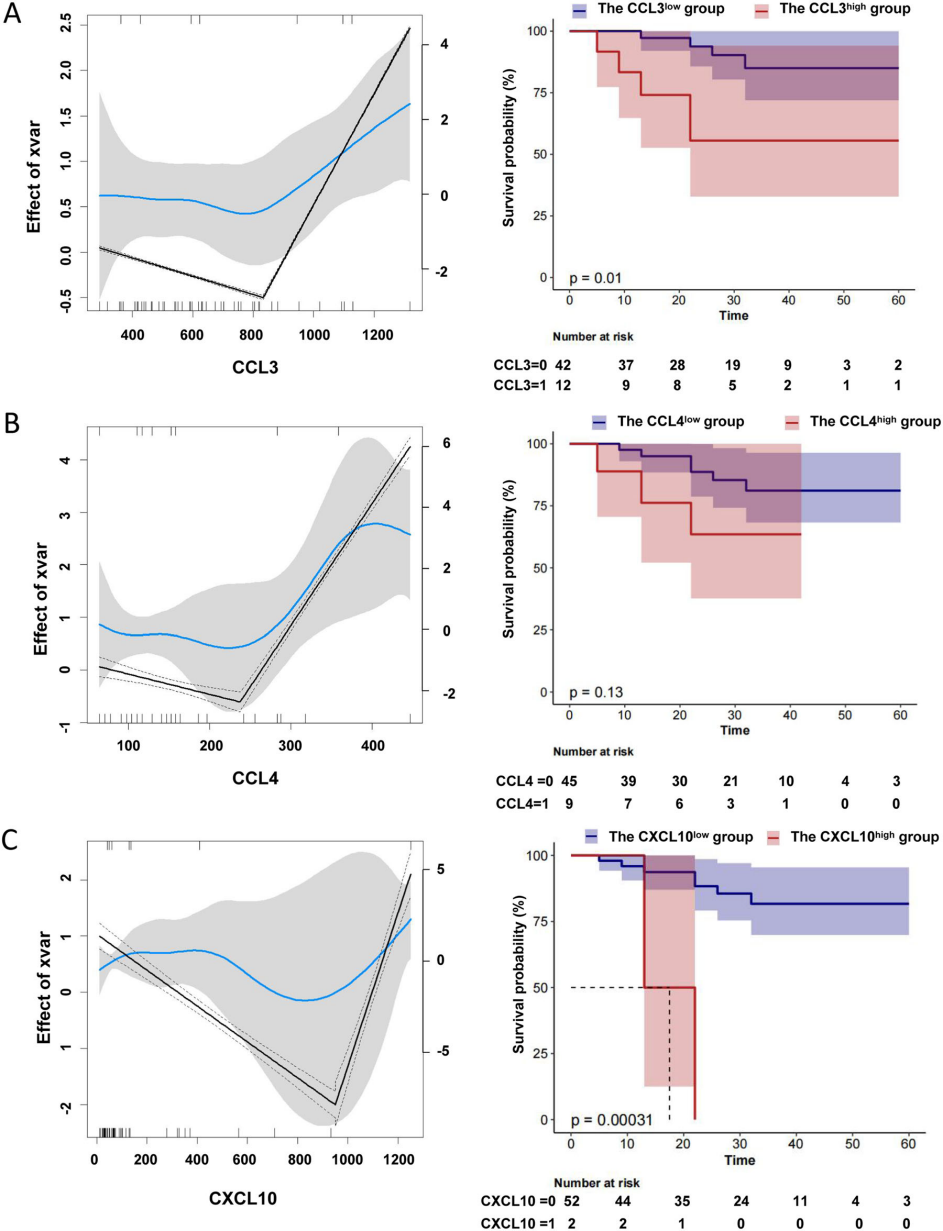
The overall survival analysis in AML patients by the cytokine protein expression. We used the statistical software packages R toolkit to find the optimal threshold of the cytokine level, and the optimal thresholds of the cytokine level were showed in the corresponding figures (left). The unit for the cytokine expression is displayed in pg/mL (X‐axes in left figure). Based on the the optimal threshold of cytokine level, the cytokines were divided into the high expression group and the low expression group. Then, the overall survival curve was assessed by Kaplan‐Meier method. The unit for follow‐up time is displayed in month (X‐axes in right figure). *p* < 0.05 was considered statistically significant.

To investigate the association of cytokine expression and the overall survival time in AML patients, K‐M curve was performed in Figure 3. We used the statistical software packages R toolkit to find the optimal threshold of the cytokine level. Based on the the optimal threshold of cytokine level, the cytokines were divided into the high expression group and the low expression group. Then, the overall survival curve was assessed by Kaplan‐Meier method. The results (Figure 3A) showed that the survival of the patients with higher CCL3 (threshold = 833.07 pg/mL) level was sinificantly shorter than those with lower CCL3 level (*p* = 0.01). A similar result was observed in the CXCL10 (Figure 3C, threshold = 949.545 pg/mL), but not in CCL4 (Figure 3B, threshold = 237.585 pg/mL), suggesting that the high expression of CCL3 and CXCL10 is associated with statistically significant reduction in overall survival of AML patients.

Additionally, although the mRNA expression level cannot fully represent the protein expression level, the data from TCGA public database was used to serve as supplementary evidence for the association between the cytokine expression and the outcome of AML patients in this study. The results showed that the survival of the patients with higher *CCL3* (threshold = 2.643) level was sinificantly shorter than those with lower *CCL3* level (Figure S1A, *p* = 0.0082). However, there were no significant differences between the level of *CCL4* (Figure S1B, threshold = −0.057) or *CXCL10* (Figure S1C, threshold = 2.834) and the outcome of AML patients.

### 3.5 The Profile of Leukocyte Subpopulations in AML Patients

**Figure 4:**
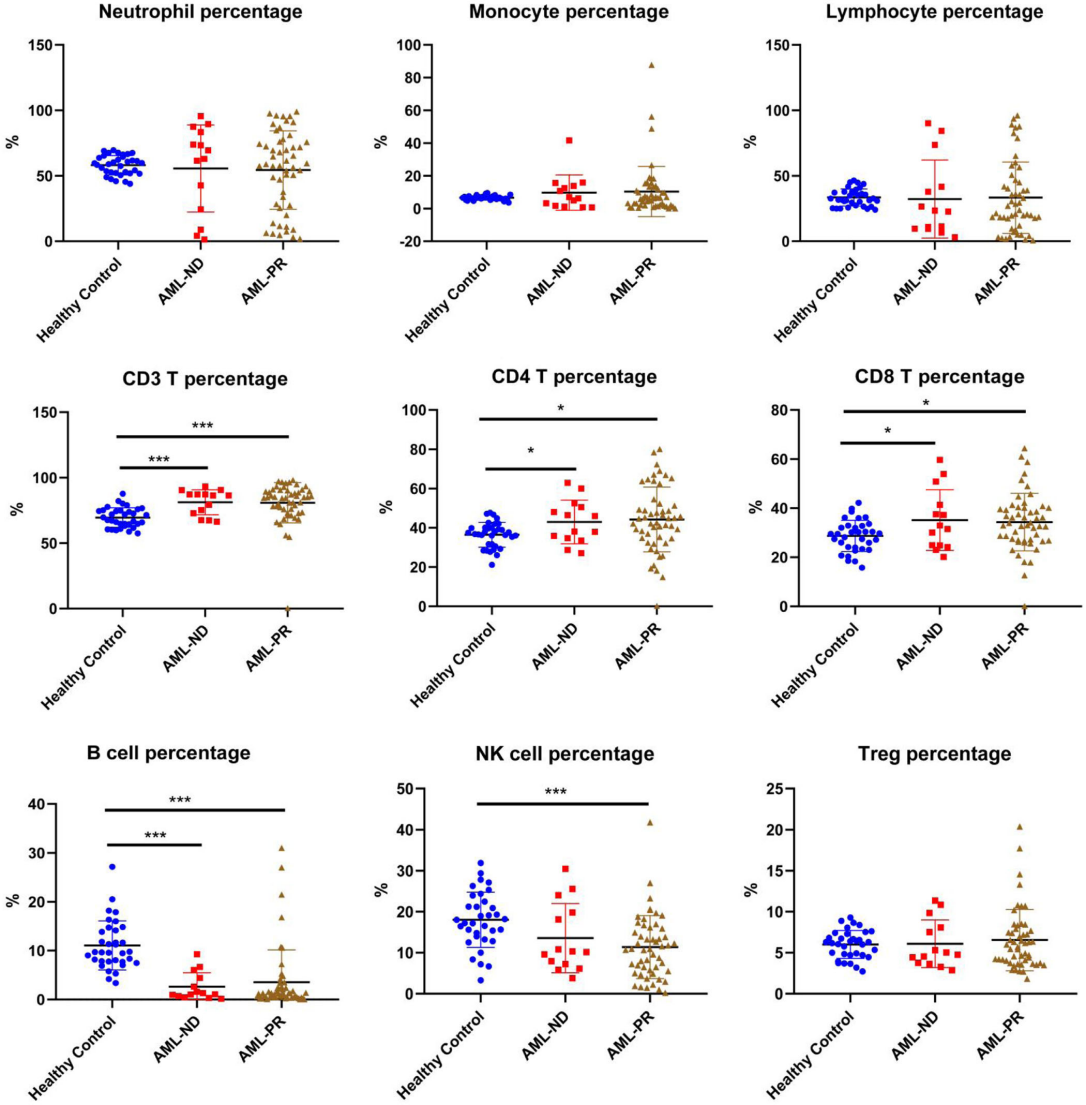
The profile of leukocyte subpopulations in AML patients. The AML patients in this assay were divided into two subgroups, the newly diagnosed group (AML‐ND) and the partial remission group (AML‐PR). The cells were labeled with antibodies to determine their phenotypes (CD3^+^ for CD3 T cells, CD3^+^ CD4^+^ for CD4 T cells, CD3^+^ CD8^+^ for CD8 T cells, CD3^‐^ CD19^+^ for B cell, CD3^‐^ CD16^+^ CD56^+^ for NK cells, and CD4^+^ C25^+^ C127^low^ for Treg cells) by flow cytometry. The neutrophils, monocytes and lymphocytes were gated according to SSC and the expression of CD45. Data in columns are shown as the mean ± SEM. (*p < 0.05, **p < 0.01, and ***p < 0.001.).

Cytokines are reported to facilitate cancer cell elimination or proliferation by interacting the body′s immunological defense mechanisms [3]. Thus, the leukocyte subpopulations were detected to investigate the relationship of peripheral mononuclear cells and the abnormally expressed cytokines in AML patients. The results of the flow cytometry showed that the frequencies of CD3 T cells, CD4 T cells and CD8 T cells in both AML‐ND and AML‐PR groups were significantly higher than those in healthy control group (Figure 4). However, the frequency of B cell in both AML‐ND and AML‐PR groups was markedly lower than that in healthy control group. Furthermore, the frequency of NK cell in AML‐PR group was lower than that in healthy control group. Additionally, there was no significant difference about the percentages of neutrophil, monocyte and lymphocyte among these groups. These results suggested that CD3 T cells, CD4 T cells, CD8 T cells, B cells and NK cells were associated with the development of AML.

Additionally, the results of absolute number showed that the numbers of all leukocyte subpopulations in both AML‐ND and AML‐PR groups were markedly lower than those in healthy control group (Figure S2), consist with the fact that the majority of peripheral mononuclear cells were leukemic cells.

### 3.6 The Correlation Between Serum Cytokines With Leukocyte Subpopulations in AML Patients

Next, the correlations between imbalanced profiles of cytokines with leukocyte subpopulations were analyzed to investigate the potential associations of the abnormal changes about the cytokines, providing clues for the source of abnormally expressed cytokines in AML microenvironment in further studies.

**Figure 5:**
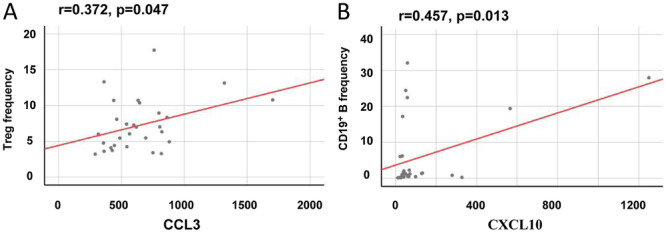
The correlation between serum cytokines with leukocyte subpopulations in AML patients. The significant correlations between CCL3 and leukocyte subpopulations were described in Figure 5A. The significant correlations between CXCL10 and leukocyte subpopulations were described in Figure 5B. Correlation analysis of continuous variables was performed by Pearson correlation analysis. *p* < 0.05 was considered statistically significant.

All correlations between imbalanced profiles of cytokines and leukocyte subpopulations were summarized in Table S2. The CCL3 level was showed to have a positive correlation with the levels of Treg cell frequency (Figure 5A). Furthermore, the serum level of CXCL10 was positively correlated with the levels of CD19^+^ B cell frequency (Figure 5B). These results suggested that Treg cells may be associated with the increase of CCL3, whereas B cells may be related to the changes of CXCL10 in AML microenvironment.

## 4 Discussion

Although there has been the great appreciation of cytokine profiles in AML microenvironment, the intricate functions of cytokines in leukemic hematopoietic microenvironment (LHME) are not fully understood. Herein, we investigated the complex cytokine networks in both AML mice and AML patients, and the imbalanced profile was significantly observed in both AML mice and AML patients. Furthermore, the inconsistent or even opposite results of cytokine expression suggest the heterogeneity among species about the imbalanced profile of cytokines in AML microenvironment. Additionally, the study showed that the high expressions of CCL3, CCL4 and CXCL10, independent of sex, age, FAB phenotype, patient status and risk molecular, were associated with the poor outcome of AML patients, suggesting they are valuable for AML prognosis. Additionally, survival analysis showed that high expression of CCL3 and CXCL10 was associated with statistically significant reduction in overall survival of AML patients. Furthermore, we tried to investigate the potential cause of abnormal changes of cytokines from the perspective of the connection between cytokines and leukocyte subpopulations. These results suggested that Treg cells may be related to the increase of CCL3, while B cells may be associated with the changes of CXCL10 in AML microenvironment.

In patients suffering from leukemic conditions, such as AML, the regulation of these cytokines is disrupted, resulting in aberrant cytokine secretion. The 9 cytokines included in the study were selected by AML mouse model or previous studies. Interestingly, the expressions of three factors were associated with the AML prognosis in this study. Consistent with the observation in AML patients in this study, high levels of CCL3 expression were reported in AML mice [7]. Moreover, suppression of Treg accumulation in the LHME and delay of leukemia progression could be achieved by blocking the CCL3‐CCR1/CCR5 axis [7], suggesting there are strong correlation between Treg cells and CCL3. High CCL4 protein level was also found in chronic lymphocytic leukemia B cells co‐cultures with nurse‐like cells, and the induction of CCL4 was abolished by a Syk inhibitor (R406), suggesting that nurse‐like cells induce the chemokine via B‐cell receptor (BCR) activation [24]. Additionally, the level of CXCL10 in the bone marrow of AML patients [25], were revealed to elevate significantly as compared to healthy individuals, and worse prognosis with higher expression of CXCL10 was observed in AML [23, 26, 27]. The upregulation of CXCL10 in B16 melanoma vessels, as well as significantly increased infiltration of CD3( + ) T‐lymphocytes in B16 tumors, was observed by inhibiting VEGF signaling in mice with B16 melanoma [28], indicating the closer links between the cytokine and lymphocytes. In brief, leukemia cells may recruit accessory cells via these chemokines, and subsequently establish a supportive microenvironment.

Although there were no significant correlations between the expressions of four cytokines (CXCL11, CXCL12, G‐CSF and sICAM‐1) with the outcome of AML in our study, these cytokine expressions in AML patients showed differences compared to those in the healthy controls in our study. The level of CXCL11 in the serum of CLL patients were revealed to elevate significantly as compared to healthy individuals [29], and worse prognosis with higher expression of CXCL11 was observed in AML [23, 26, 27]. In addition, CXCL12, also known as stromal derived factor‐1 (SDF‐1), is essential for immune system foundation, hematopoietic cell development, migration and homing of hematopoietic stem cells to the BM via its receptor CXCR4. Consist with this study, there was a significantly decreased expression of CXCL12 in AML. Additionally, the low level of SDF‐1 transcripts in bone marrow mesenchymal stem cells of patients with AML might be disadvantageous on the engraftment after HSCT [30], which was similar with the our result that the expression of CXCL12 in HSCT patients was significantly higher than those in AML‐ND and AML‐PR group, but not healthy controls, indicating that the cytokine microenvironment in AML patients who had reestablished hematopoiesis at least 6 months after allo‐HSCT was similar with the status in healthy population. Additionally, the hematopoietic growth factor G‐CSF, available as recombinant products, stimulates the growth in culture of blasts from AML patients [31]. Complete remission was observed in three patients with AML by administration of G‐CSF without the use of anti‐leukemic agents, suggesting the possibility that G‐CSF itself has the potential to lead some AML patients to complete remission [32]. Furthermore, the serum levels of sICAM‐1 were increased and showed an association with the clinical stage and prognostic markers in chronic B‐lymphocytic leukemia [33].

Additionally, the expressions of two cytokine (IL‐6 and IL‐5) did not show any significant differences compared to the healthy controls. Similar with our findings, decreased level of IL‐16 were observed after Ibrutinib treatment in those patients with chronic lymphocytic leukemia [34], emphasizing that IL‐16 is correlated with disease burden and aberrant immune microenvironment in leukemia patients. The activity of interleukin‐5 (IL‐5) is known as a growth factor for eosinophils, and overexpression of IL‐5 was observed in a chronic eosinophilic leukemia murine model [35]. However, only the expression level of IL5 was not observed to have a significant change in this study, suggesting IL‐5 may have a limited effect on AML microenvironment.

There are also certain limitations in our study. Firstly, the reliance on pathogen‐free mice with a specific inbred background likely skews the observed immune response to AML in this AML mouse model. This approach inherently limits the generalizability of findings to humans, as the immune systems of these mice are not representative of the diverse and dynamic human immune responses. Consequently, using these data about inflammatory cytokines in humans introduces potential bias. Moreover, the AML patients were highly heterogeneous including different tumor‐driving genetic alterations or FAB phenotype. However, only the AML mouse model with MLL‐AF9 genetic mutation was investigated in this study., *e.g*. the inconsistent or even opposite results of cytokine expression in AML mice and AML patients, suggesting the presence of potential bias due to different species or genetic background. Secondly, only 4 cytokines from the 22 observed to be lower in the spleen and bone marrow of AML mice, based on a comprehensive assessment including literature evidence, expression magnitude, and clinical feasibility, were selected for the subsequent AML patient assay, resulting in the potential selection bias. Thirdly, although FAB phenotype, patient status and risk molecular, which may affect prognosis of AML patients, were analyzed in the relation analysis with the outcome of AML patients, no significant difference was observed due to the small sample size, especially for the patients undergoing allo‐HSCT (*n* = 7), or the M3 patients (*n* = 1), resulting in potential bias. This needs to be verified in further studies with a larger sample size overcoming the difficulties in patient source. Fourthly, although the bone marrow or spleen are the optimal microenvironment for hematopoiesis, the focus of our research was to investigate the possibility of using cytokines in peripheral blood as clinical predictive indicators in AML. Consequently, experiments sourced from bone marrow or spleen in AML patients were not designed in this study. Additionally, despite the correlation between cytokines and immune cell subpopulations discussed in the study, the change of cytokines may be causes or consequences of the change of immune subpopulations in the AML microenvironment, and more mechanistic studies are needed to clarify the potential possibility.

## 5 Conclusion

In summary, we investigated the complex cytokine networks in both AML mice and AML patients, and the imbalanced profile was significantly observed. Additionally, the study showed that the high expressions of CCL3, CCL4 and CXCL10 were associated with the poor outcome of AML patients, suggesting they are valuable for AML prognosis. Moreover, the correlation between serum cytokines with leukocyte subpopulations in AML patients suggested that Treg cells may be related to the increase of CCL3, while B cells may be associated with the changes of CXCL10 in AML microenvironment. The change of leukocyte subpopulations, either as causes or as consequences, may partially account for the change of cytokines in AML condition. The distinct patterns of cytokines in peripheral blood circulation, may shape both the BM microenvironment and hematopoietic processes, eventually leading to the development of AML.

## Author Contributions

**Rong Wang:** data curation, formal analysis, methodology, project administration, validation, and writing – original draft. **Keying Jing:** data curation, methodology, project administration, software, and writing – review and editing. **Huijuan Zhao:** formal analysis, investigation, supervision, and writing – review and editing. **Guoguang Zheng:** conceptualization, supervision, visualization, and writing – review and editing. **Jun Cai:** conceptualization, funding acquisition, resources, supervision, and writing – review and editing.

## Conflicts of Interest

The authors declare no conflicts of interest.

## Supporting information

Figure S1. The overall survival analysis in AML patients by the cytokine mRNA expression.The TCGA public database was used for the analysis of overall survival (OS) in acute myeloid leukemia patients (TCGA, NEJM 2013, PMID: 23634996). We used the statistical software packages R toolkit to find the optimal threshold of the cytokine level, and the optimal thresholds of the cytokine level were showed in the corresponding figures (left). Based on the the optimal threshold of cytokine level, the cytokines were divided into the high expression group and the low expression group. Then, the overall survival curve was assessed by Kaplan‐Meier method. *P* < 0.05 was considered statistically significant.

FigureS2. The gating strategies for all subpopulation cells in this work. The gating strategies for neutrophils, monocytes, lymphocytes were showed according to CD45 expression and SSC. Gated on the population of lymphocytes, NK cells were defined as CD3‐ CD16+ CD56+ cells, B cells were defined as CD3‐ CD19+ cells, CD3 T cells were defined as CD3+ T cells, CD4 T were defined as CD3+ CD4+ CD8‐ T cells, and CD8 T were defined as CD3+ CD4‐ CD8+ T cells. The gating strategies were described in Figure S2A. Additionally, the gating strategies for Treg cells were described in Figure S2B. Treg cells were defined as CD3+ CD4+ CD25+ CD127low cells based on the population of lymphocytes. Fluorescence Minus One (FMO) control for the CD127 PE (CD127 Negitive Control) and CD25 APC (CD25 Negitive Control) plot, gated on the CD3+ CD4+ lymphocytes, were also presented in Figure S2B.

Figure S3. The absolute number of leukocyte subpopulations in AML patients. The AML patients in this assay were divided into two subgroups, the newly diagnosed group (AML‐ND) and the partial remission group (AML‐PR). Data in columns are shown as the mean ± SEM. (*p < 0.05, **p < 0.01, and ***p < 0.001.

Supplement Table‐S1‐2.

## Data Availability

I confirm that my article contains a Data Availability Statement even if no data is available (list of sample statements) unless my article type does not require one (e.g., Editorials, Corrections, Book Reviews, etc.).
