# Supplementary figures and images for: The Imbalanced Patterns and Clinical Significance of Cytokines in Acute Myeloid Leukemia Microenvironment
Source: Immun Inflamm Dis. 2025 Nov 10;13(11):e70290. doi: 10.1002/iid3.70290 (PMC12598404; doi:10.1002/iid3.70290)

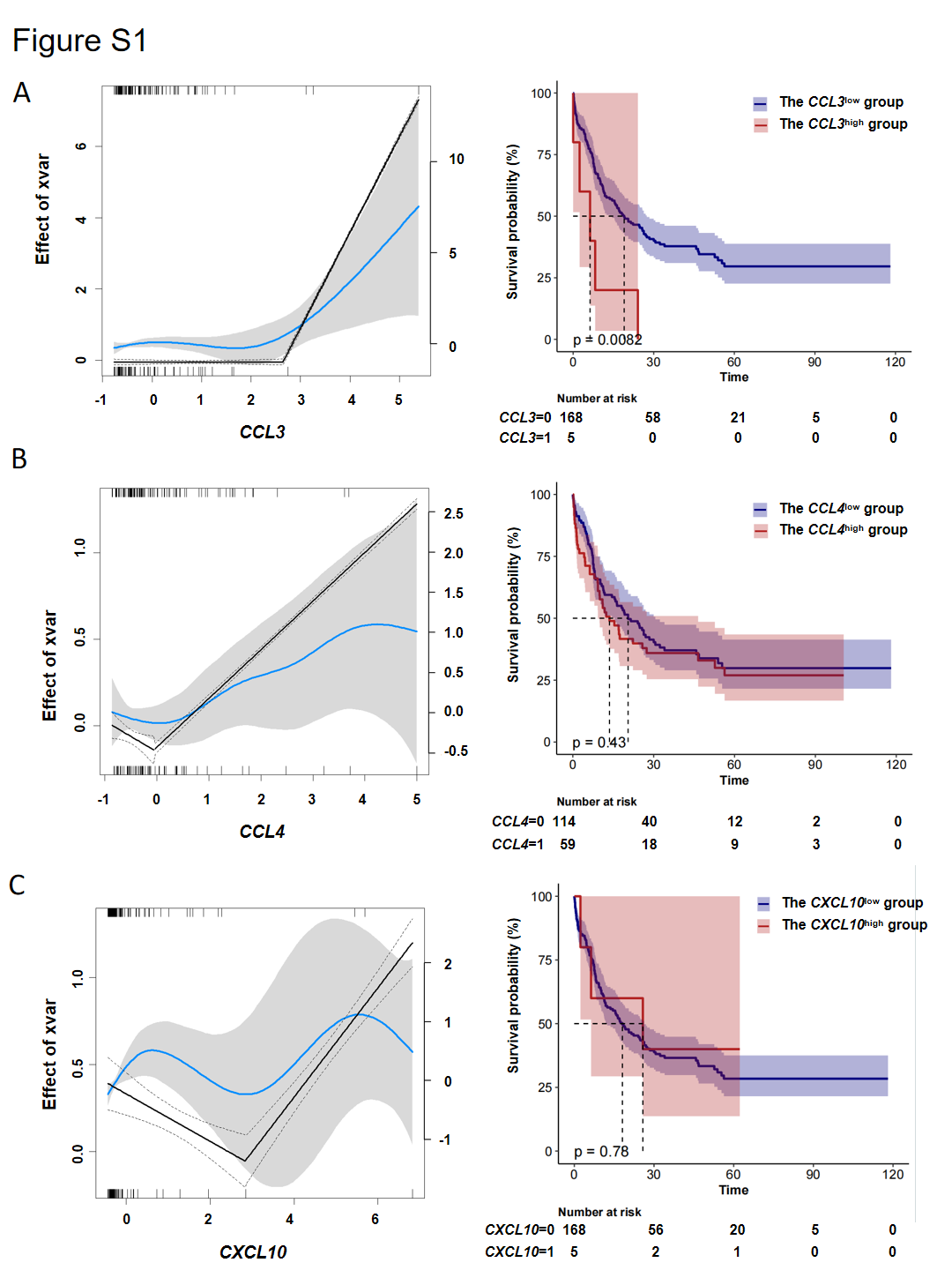

Supplement: Supplementary file 1 — Figure S1. The overall survival analysis in AML patients by the cytokine mRNA expression.The TCGA public database was used for the analysis of overall survival (OS) in acute myeloid leukemia patients (TCGA, NEJM 2013, PMID: 23634996). We used the statistical software packages R toolkit to find the optimal threshold of the cytokine level, and the optimal thresholds of the cytokine level were showed in the corresponding figures (left). Based on the the optimal threshold of cytokine level, the cytokines were divided into the high expression group and the low expression group. Then, the overall survival curve was assessed by Kaplan‐Meier method. P < 0.05 was considered statistically significant. [file IID3-13-e70290-s003.tif]

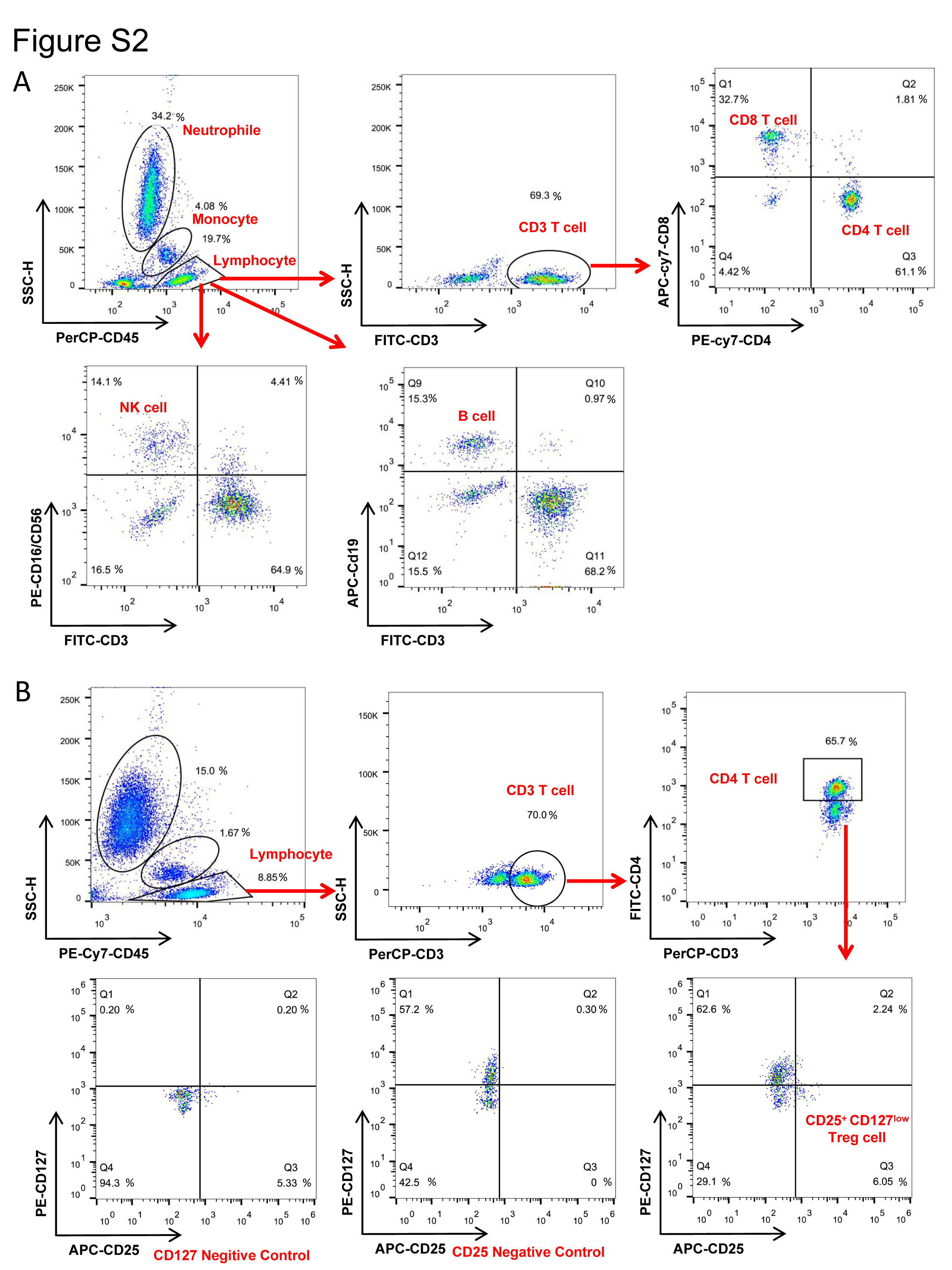

Supplement: Supplementary file 2 — FigureS2. The gating strategies for all subpopulation cells in this work. The gating strategies for neutrophils, monocytes, lymphocytes were showed according to CD45 expression and SSC. Gated on the population of lymphocytes, NK cells were defined as CD3‐ CD16+ CD56+ cells, B cells were defined as CD3‐ CD19+ cells, CD3 T cells were defined as CD3+ T cells, CD4 T were defined as CD3+ CD4+ CD8‐ T cells, and CD8 T were defined as CD3+ CD4‐ CD8+ T cells. The gating strategies were described in Figure S2A. Additionally, the gating strategies for Treg cells were described in Figure S2B. Treg cells were defined as CD3+ CD4+ CD25+ CD127low cells based on the population of lymphocytes. Fluorescence Minus One (FMO) control for the CD127 PE (CD127 Negitive Control) and CD25 APC (CD25 Negitive Control) plot, gated on the CD3+ CD4+ lymphocytes, were also presented in Figure S2B. [file IID3-13-e70290-s002.tif]

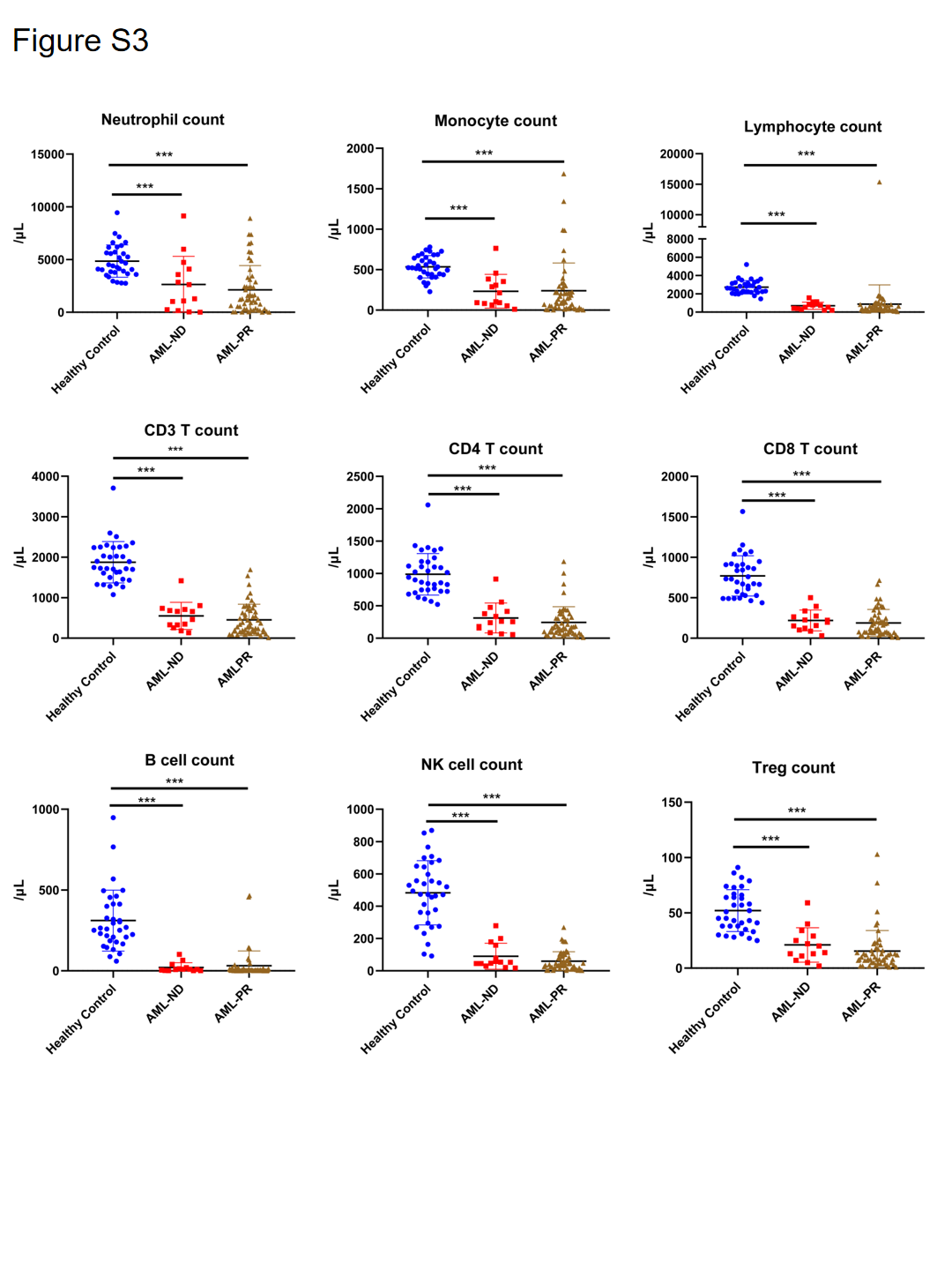

Supplement: Supplementary file 3 — Figure S3. The absolute number of leukocyte subpopulations in AML patients. The AML patients in this assay were divided into two subgroups, the newly diagnosed group (AML‐ND) and the partial remission group (AML‐PR). Data in columns are shown as the mean ± SEM. (*p < 0.05, **p < 0.01, and ***p < 0.001. [file IID3-13-e70290-s001.tif]
