# Supplementary material for: The Imbalanced Patterns and Clinical Significance of Cytokines in Acute Myeloid Leukemia Microenvironment
Source: Immun Inflamm Dis. 2025 Nov 10;13(11):e70290. doi: 10.1002/iid3.70290 (PMC12598404; doi:10.1002/iid3.70290)
Supplement: Supplementary file 4 — Supplement Table‐S1‐2. [file IID3-13-e70290-s004.docx]

**Table S1. Summary of changes about the cytokine panel in AML mice**

| **Kinds of change** | **cytokine** |
| --- | --- |
| SP-up and BM-up (5) | CXCL11、G-CSF、IL-16、IL-5、sICAM-1 |
| SP-up and BM-0 (2) | IL-13、TIMP-1 |
| SP-up and BM-down (7) | TREM-1、C5/C5a、IL-12 p70、IL-3、IL-4、IL-6、GM-CSF |
| SP-0 and BM-0 (2) | IL-1α、IFN-γ |
| SP-down and BM-up (1) | KC/CXCL1 |
| SP-down and BM-0 (1) | IL-2 |
| SP-down and BM-down(22) | IL-17、CCL1、CCL11、CCL2/MCP-1、CXCL10、CXCL13、IL-10、IL-1β、IL-1rα、IL-7、IL-23、IL-27、MCP-5/CCL12、M-CSF、MIG/CXCL9、MIP-1α/CCL3、MIP-1β/CCL4、MIP-2/CXCL2、RANTES/CCL5、SDF-1/CXCL12、TARC/CCL17、TNF-α |

SP, spleen; BM, bone marrow, G-CSF, granulocyte colony-stimulating factor; sicam-1, soluble intercellular adhesion molecular-1; TIMP-1, tissue inhibitor of metalloproteinase-1; TREM-1, triggering receptor expressed on myeloid cells-1; GM-CSF, granulocyte-macrophage colony-stimulating factor; M-CSF, macrophage colony-stimulating factor; A total of 40 cytokines were described in the table.

**Table S2.** **Summary of correlations between cytokine level and cell subpopulation in AML patients**

| Cytokines | Neutrophil | | Monocyte | | Lymphocyte | | CD3^+^ T cell | | CD4^+^ T cell | |
| --- | --- | --- | --- | --- | --- | --- | --- | --- | --- | --- |
|  | Coeff.(95%CI) | p | Coeff.(95%CI) | p | Coeff.(95%CI) | p | Coeff.(95%CI) | p | Coeff.(95%CI) | p |
| CXCL10 | 0.02 (-0.03,0.07) | 0.425 | 0 (-0.01,0.01) | 0.626 | -0.02 (-0.07,0.03) | 0.392 | -0.02 (-0.04,0) | 0.096 | -0.01 (-0.04,0.01) | 0.357 |
| IL-16 | 0.02 (-0.13,0.17) | 0.753 | -0.01 (-0.03,0.02) | 0.667 | -0.02 (-0.17,0.13) | 0.81 | -0.04 (-0.1,0.02) | 0.194 | -0.01 (-0.08,0.05) | 0.698 |
| CCL3 | -0.01 (-0.05,0.03) | 0.468 | 0 (-0.01,0.01) | 0.887 | 0.01 (-0.03,0.05) | 0.457 | 0 (-0.02,0.02) | 0.969 | 0 (-0.02,0.03) | 0.665 |
| CCL4 | 0.02 (-0.11,0.14) | 0.784 | 0.01 (-0.01,0.03) | 0.247 | -0.03 (-0.15,0.1) | 0.652 | 0.02 (-0.04,0.08) | 0.588 | 0.06 (-0.01,0.12) | 0.081 |
| G-CSF | 0 (0,0) | 0.648 | 0 (0,0) | 0.864 | 0 (0,0) | 0.65 | 0 (0,0) | 0.153 | 0 (0,0) | 0.015 |
| sICAM-1 | 0 (0,0) | 0.04 | 0 (0,0) | 0.805 | 0 (0,0) | 0.038 | 0 (0,0) | 0.03 | 0 (0,0) | 0.106 |
| CXCL11 | -0.07 (-0.13,-0.01) | 0.033 | -0.01 (-0.02,0) | 0.166 | 0.08 (0.02,0.14) | 0.017 | 0.03 (-0.01,0.06) | 0.139 | 0.03 (-0.01,0.07) | 0.121 |
| CXCL12 | 0.01 (-0.01,0.03) | 0.264 | 0 (0,0) | 0.389 | -0.01 (-0.02,0.01) | 0.357 | -0.01 (-0.01,0) | 0.089 | -0.01 (-0.01,0) | 0.203 |
| IL-5 | 0.74 (-4.64,6.13) | 0.776 | 0.39 (-0.51,1.29) | 0.376 | -1.18 (-6.58,4.23) | 0.655 | -1.04 (-3.82,1.73) | 0.448 | -1.89 (-4.82,1.05) | 0.198 |

| Cytokines | CD8^+^ T cell | | CD19^+^ B cell | | NK cell | | Treg cell | |
| --- | --- | --- | --- | --- | --- | --- | --- | --- |
|  | Coeff.(95%CI) | p | Coeff.(95%CI) | p | Coeff.(95%CI) | p | Coeff.(95%CI) | p |
| CXCL10 | -0.01 (-0.03,0.01) | 0.236 | 0.02 (0,0.03) | 0.013 | 0 (-0.01,0.02) | 0.783 | 0 (0,0.01) | 0.162 |
| IL-16 | -0.03 (-0.07,0.02) | 0.23 | 0.04 (0,0.07) | 0.056 | 0.01 (-0.03,0.05) | 0.709 | -0.01 (-0.02,0.01) | 0.339 |
| CCL3 | -0.01 (-0.02,0.01) | 0.218 | 0 (-0.01,0.01) | 0.872 | 0 (-0.01,0.01) | 0.975 | 0 (0,0.01) | 0.047 |
| CCL4 | -0.04 (-0.08,0) | 0.057 | -0.01 (-0.05,0.03) | 0.679 | -0.01 (-0.05,0.03) | 0.513 | 0 (-0.02,0.01) | 0.771 |
| G-CSF | 0 (0,0) | 0.21 | 0 (0,0) | 0.258 | 0 (0,0) | 0.282 | 0 (0,0) | 0.342 |
| sICAM-1 | 0 (0,0) | 0.631 | 0 (0,0) | 0.12 | 0 (0,0) | 0.14 | 0 (0,0) | 0.74 |
| CXCL11 | 0 (-0.03,0.03) | 0.898 | -0.01 (-0.03,0.02) | 0.491 | -0.02 (-0.04,0.01) | 0.138 | 0 (-0.01,0.01) | 0.53 |
| CXCL12 | 0 (-0.01,0) | 0.549 | 0 (0,0.01) | 0.024 | 0 (0,0.01) | 0.449 | 0 (0,0) | 0.451 |
| IL-5 | -0.24 (-2.25,1.77) | 0.806 | 0.15 (-1.58,1.88) | 0.861 | 0.87 (-0.84,2.58) | 0.306 | -0.38 (-0.99,0.24) | 0.219 |

Coeff, coefficients; Data presented are coefficients and 95% CIs. Correlation analysis of continuous variables was performed by Pearson correlation analysis. P < 0.05 was considered statistically significant.
